# A Review on Iron Chelators in Treatment of Iron Overload Syndromes

**Published:** 2016-10-01

**Authors:** Naser Mobarra, Mehrnoosh Shanaki, Hassan Ehteram, Hajar Nasiri, Mehdi Sahmani, Mohsen Saeidi, Mehdi Goudarzi, Hoda Pourkarim, Mehdi Azad

**Affiliations:** 1 Metabolic Disorders Research Center, Department of Biochemistry, School of Medicine, Golestan University of Medical Sciences, Gorgan, Iran; 2 Department of Laboratory Medicine, School of Allied Medical Sciences, Shahid Beheshti University of Medical Sciences, Tehran, Iran; 3 Department of Pathology, School of Medicine, Kashan University of Medical Sciences, Kashan, Iran; 4 Hematology-Oncology and Stem Cell Transplantation Research Center, Tehran University of Medical Sciences, Tehran, Iran; 5 Department of Clinical Biochemistry, Cellular and Molecular Research Center, Qazvin University of Medical Sciences, Qazvin, Iran; 6 Stem Cell Research Center, Golestan University of Medical Sciences, Gorgan, Iran; 7 Department of Microbiology, School of Medicine, Shahid Beheshti University of Medical Sciences, Tehran, Iran; 8 Department of Hematology, Allied Medical School, Tehran University of Medical Sciences, Tehran, Iran; 9 Department of Medical Laboratory Sciences, Faculty of Allied Medicine, Qazvin University of Medical Sciences, Qazvin, Iran

**Keywords:** Chelators, Iron overload, Treatment

## Abstract

Iron chelation therapy is used to reduce iron overload development due to its deposition in various organs such as liver and heart after regular transfusion. In this review, different iron chelators implicated in treatment of iron overload in various clinical conditions have been evaluated using more up-to-date studies focusing on these therapeutic agents. Deferoxamine, Deferiprone and Deferasirox are the most important specific US FDA-approved iron chelators. Each of these chelators has their own advantages and disadvantages, various target diseases, levels of deposited iron and clinical symptoms of the afflicted patients which may affect their selection as the best modality. Taken together, in many clinical disorders, choosing a standard chelator does not have an accurate index which requires further clarifications. The aim of this review is to introduce and compare the different iron chelators regarding their advantages and disadvantages, usage dose and specific applications.

## Introduction

 Iron is one of the essential elements in body which its concentration is tightly regulated. Iron overload during iron deposition in multiple organs is along with serum ferritin value over than 1000 µg/L.^   1 ^ Iron overload, either genetically or acquired, may occur by several conditions such as frequent transfusions, abuse consumption of iron (often as supplement) and chronic hepatitis have potential to cause acquired iron overload.^   2 ^ ^-^3  Among genetic disorders that causes iron overload including hereditary hemochromatosis (all types), African iron overload, sickle cell disease, major beta-thalassemia, sideroblastic anemia, enzyme deficiency (pyruvate kinase, G6PD) and rare disorders of transporting proteins (Atransferrinemia, Aceruloplasminemia), ^3^^-^^8^  hereditary hemochromatosis is the most common genetic causes of iron overload.^   9 ^ Small intestine in patient absorbs high level iron which accumulates in liver, pancreas and some parts of brain which results to impair vital functions.^   10 ^ Free radical production due to iron overload causes serious complicated side effects such as mental retardation,^        11 ^ early neurological diseases (Alzheimer's, multiple sclerosis, Huntington),^        12 ^ delays in sexual maturity,^   13 ^ impotence and infertility,^   14 ^ cardiac dysfunction (arrhythmia, cardiomyopathy, hemosiderosis),^        15 ^ liver cirrhosis, liver cancer and hepatitis^   16 ^ and metabolism dysfunction (diabetes, hypogonadism, thyroid disorders, parathyroid and less level of adrenal glands).^   17 ^ The others include arthritis, chronic fatigue, depression, hair loss, skin color changing, abdominal pain, splenomegaly, infection with HIV, venous thrombosis and osteoporosis.^        18 ^

71% mortality in cardiac disease due to iron accumulation in myocardium is a significant complication of iron overload in beta-thalassemia. ^11^^,^^        19 ^ In order to avoid serious complications of iron overload, it is essential to suppress LPI (Labile Plasma Iron) and remove excess iron.^        20 ^ In major beta-thalassemia and hereditary hemochromatosis, phlebotomy is impossible because patients are anemic. Thus, the best selection for treatment of iron overload is iron chelation therapy.^   21 ^ The history of chelation therapy goes back to early 1930s when Ferdinand Mans worked out on synthesis of ethylene diamine tetra-acetic acid (EDTA). 3^,^^   22 ^ Afterwards, researchers found that EDTA is effective in treatment of lead poisoning.^        23 ^ ^-^^24^  From 1970s, chelation therapy was replaced by phlebotomy to remove excess iron in patients with hemochromatosis.^        18 ^ Chelators are able to bind metal ions for drastic reduction in their reactivity.^   25 ^ The ultimate complex is water soluble which can enter bloodstream and excrete without any damages. However, there was cardiac arrest during treatment with chelation due to hypocalcemia.^   26 ^ In 2005, a five-year-old boy with autism and a three-year-old girl as well as an adult with no signs of autism passed away during chelation therapy.^        27 ^ It seems that in two hypocalcemia cases, using disodium-EDTA was reason but in the third case, the type of EDTA was unknown. According to the three-year-old girl medical record, increasing level of lead in her blood can be cause of her anemia.^        28 ^ Nowadays, using EDTA in not common for children.^   29 ^

**Common methods in evaluation of Iron Overload**

There are various different methods for evaluating iron overload degree including serum ferritin levels, liver iron concentration determined from biopsy, superconducting quantum interference device (SQUID) and magnetic resonance imaging (MRI). Each method has pros and cons to quantify and monitor iron burden.^        30 ^ The simplest way to quantify iron overload is to measure serum ferritin level which correlates with iron stores in body. However, ferritin level is variable in the presence of inflammation or infection and vitamin C deficiency; as a result the reliability of this method is questionable.^   31 ^ The best way to assess iron overload is liver biopsy, but it is invasive for screening. Liver iron concentration above 15 mg/gram of dry weight predicts a higher risk of cardiac disease and progression of hepatic fibrosis.^   32 ^ MRI is a non invasive method for evaluation of hepatic and cardiac iron to replace by liver biopsy (T1, T2, T2* are MRI parameters that reduced in iron overload). In addition, cardiac iron overload in asymptomatic patients characterized by T2 and MRI signal changing evaluate response to chelation therapy. Recently, we evaluate cardiac and liver iron by MRI because it is useful as a preliminary assessment on clinical chelation therapy.^   33 ^

The risk of left ventricular dysfunction is increased in patients with major beta-thalassemia due to iron overload. In patients with low levels of T2 (less than 6 ms), the progression of cardiac failure related to iron density is 47%. In cardiac disorder or arrhythmia, chelation therapy process should be intensified; thus, it is recommended to use a combination of iron chelators.^        34 ^

Iron is removed from different organs at different rates: hepatic iron burden usually improves more rapidly than cardiac iron burden with intensification of chelation. Therefore, both hepatic and cardiac iron must be measured to optimize chelation therapy. Ferritin level changes paralleled with liver iron concentration variations.^        35 ^ In particular, ferritin levels above 2,500 ng/mL are associated with an increased risk of morbidity and mortality and should be trigger intensification of chelation therapy.^   36 ^

**Iron Chelation therapy**

The aim of chelation therapy is to prevent the accumulation of excess iron and its complication such as hepatic, endocrinological and cardiac dysfunction.^   37 ^

The evidence show that the initiating stage of chelation therapy in patients with major beta-thalassemia is a key factor in their survival.^3^ This stage includes an alternation in ferritin level to above 1000 ng/mL. Thus, effective chelation therapy period trapped non-transferrin bound iron and LPI to prevent adverse complications of iron overload.^   38 ^ Several iron chelators have been designed to excrete tissue iron through urine or feces by forming complexes.^   39 ^

**Different types of Iron Chelators and review of their generalities**

**1. DEFEROXAMINE**

 (DFO or Desferal) is a non-toxic iron chelator which is clinically approved and effective for long-term iron chelation therapy in beta-thalassemia and other iron overload cases. Remarkable effect of DFO on reduction of serum ferritin level and hepatic iron is inevitable which increases longevity.^        40 ^ Despite of DFO oral absorption ability, pharmacokinetics of oral forms of chelators is not optimal. Ineffectively of its intramuscular injection also has been proven. Therefore, it is not used orally or IM and continuous intravenous or subcutaneous infusion should be recommended.^   41 ^

The main mechanisms of iron deposition by DFO are as follows:^        42 ^

1. DFO is a hexadentate chelator, binding iron at a 1:1 molar ratio.
2. Old RBC iron storage will be released by reticuloendothelial system macrophages and precipitated by DFO and rapidly excreted through urine.
3. Non-bonded DFO will be internalized by liver parenchymal cells and attached to excess hepatic iron and excreted via bile.
4. DFO can directly absorb iron accumulation in cardiac muscle cells.

Due to DFO short plasma half-life, continuous injection is required for patient with iron overload until iron level disposal reaches to 15 mg daily. As a result of nocturnal injection of Deferoxamine, 20 to 50 mg of iron (600 to 1,500 mg per month) should be excreted through urine and feces daily. Therefore, it can minimize iron reaccumulation and decrease its storage if the transfusion is less than 4 packed RBC per month (less than 800 mg of iron).^        42 ^ Although the treatment with DFO is effective, its infusions are time-consuming, expensive and painful in children. Moreover, they frequently have a negative impact on patient's quality of life.^        43 ^

Dose-dependent side effects of Deferoxamine are visual and auditory neurotoxicity due to chronic treatment and acute effects including abdominal pain, diarrhea, nausea, vomiting and hypotension. Accordingly, annual testing by optometrists and audiometerists is recommended.^42^^,^^        43 ^

Fortunately, most toxicity is reversible when DFO treatment is withdrawn. Treatment with high doses of DFO is associated with blood pressure increase in lungs.^        44 ^ Deferoxamine therapy increases the risk of infection of mucormycosis, vibrio and yersinia. It should be mentioned that it cannot be seen with other iron chelators such as Deferasirox and Deferiprone because they do not work as siderophores.^   45 ^

**2. DEFERIPRONE**

Oral iron chelator which is proper choice for patients who showed an inadequate response to prior chelation therapy such as Deferasirox and Deferoxamin. The most typical side effects include elevated liver enzymes, gastrointestinal disorders and arthralgia. The most serious adverse effects associated with DFP are agranulocytosis and neutropenia with an incidence of 0.2 and 2.8 per 100 patients over one year that are reversible after stopping therapy.^37^^,^^46^ 

A major problem in Deferiprone therapy in hepatic cell culture studies (with iron accumulation) demonstrated that increased oxidative DNA damage occurs when chelator ratio is lower than iron concentration.^   47 ^ The primary recommended oral dose of Deferiprone is 25 mg/kg 3 times a day (daily consumption: 75 mg/kg) and a maximum recommended daily use is 100 mg/kg. For agranulocytosis and neutropenia monitoring during therapy, neutrophil absolute count should be performed regularly.^   48 ^ In comparison with Deferoxamine and Deferasirox, it seems that deferiprone is not successful enough in controlling iron overload in thalassemia. Despite of its high percent (79%-80%) acceptance compared to deferoxamine (59%-78%), its efficiency and immunity remains questionable. Deferiprone is used as the second choice treatment in major beta-thalassemia patients when deferoxamine is not available. ^   49 ^

Basically, in iron overload condition such as hereditary hemochromatosis, deferiprone therapy would be intolerable. Also, transfusion dependent patients with severe conflict cardiac require more serious chelation therapy than regular patients with chelation therapy. In such cases, combination therapy with subcutaneous or intravenous deferoxamine and oral deferipronem is recommended. This combination therapy will be significantly improve severe cardiac siderosis or left ventricular dysfunction.^46^^-^^   49 ^

**3. DEFERASIROX**

Tridentate iron chelating agent that binds iron in a 2:1 ratio. This combination has high affinity for iron and very low affinity for copper and zinc.^        50 ^ The most common side effects of DFX are abdominal pain, nausea, vomiting, diarrhea, skin rashes and ophthalmic complication. These reactions frequently occur in older patients with predisposition to myelodysplastic syndrome, renal or hepatic disease and patients with low platelet counts.^   51 ^ Serum creatinine level, serum transaminases, bilirubin and CBC should be regularly monitored. One of troublesome factors in Deferasirox therapy is proximal renal tubular dysfunction^   52 ^ and other complications including drastic level of metabolic acidosis, hypophosphatemia and hypokalemia.

In study from Al-Khabori, et al.^52^ Deferasirox withdrawal and replacement therapy in 4 patients with similar conditions rapidly resulted in normal balance of electrolytes. It is worth noting that in order to prevent these complications; the patients should avoid the use of antacid containing aluminum such as Maalox and Mylanta during Deferasirox therapy.^53^

**Formulation of Chelators and their consumption dosage**

Deferiprone is presented as 500 mg tablets and oral solution (100 mg in 0.4 ml). The common dosage is 75-100 mg/kg/day in 3 doses per day. Deferasirox is available in different tablet sizes such as 125 mg, 250 mg and 500 mg. They are completely water or juice soluble and can be taken with an empty stomach. The recommended dosage is 20 mg/kg up to a maximum of 30 mg/kg/day in a single dose. Ferritin level monitoring will prescribe the further dosage.^   54 ^

Expenses of Deferiprone and Deferasirox therapy can be compared from different points of view: medication prices, laboratory tests cost and side effects related treatment cost.^   4 ^ It is estimated that Deferiprone therapy cost for a child could be twice higher than Deferoxamine therapy. As a result, Deferasirox is the first choice and then both Deferiprone and Deferoxamine surge into second and third place*, *respectively. However, this comparison can still be various in different countries. Several recent studies indicate that Deferasirox therapy is more affordable than traditional Deferoxamine therapy (Table 1).^   54 ^

**Relative and actual Chelation therapy expenses**

Before appearance of iron chelation therapy, there were complications in chronically transfused patients such as cardiomyopathy, hepatic cirrhosis, endocrine disorders and early death.^   55 ^ Deferoxamine was the first iron chelator which gets FDA approval in 1968. DFO significantly reduces iron burden and prevents a large portion of life-threatening iron overload complications. The first clinical use of oral DFO therapy in human began in 1987. However, the cost of Desferal treatment is too high.^        56 ^ Due to short plasma half-life of Desferal, it must be given continuously over 8-12 hours, 5-7 days/week. Accordingly, the burden of this regimen is often difficult and this leads to development o iron overload complications and related therapeutic expenses.

**Table 1:** General properties of Iron Chelators

**Property**	**Deferasirox**	**Deferiprone**	**Deferoxamine**
**Route**	Oral	Oral	Subcutaneous, intravenous
**Usual dose**	20-30 mg/kg/day	75 mg/kg/day	25-50 mg/kg/day
**Schedule**	Once a day	in 3 divided doses daily	day over 8-24 hours, 5 days week
**Side effect**	Gastrointestinal Renal failure	Gastrointestinal Agranulocytosis/ neutropenia Arthralgia	Rash Ophthalmological Auditory
**Advantage**	Oral and daily use	Effective in cardiac iron excretion	availability of information
**Disadvantage**	unavailability of complete information	blood count monitoring	Significant toxicity

Meanwhile, Desferal is an expensive medication by itself and treatment cost will be doubled.^        57 ^ Due to its serious side effects regular monitoring of visual system, hearing system and physical abilities in patients are necessary. Regular measurement of ferritin levels, liver biopsy, SQUID or new MRI technology called FerriScan can be helpful. As SQUID is not yet a worldwide technology, traveling expenses may impose additional costs on patients. Based on these problems, the oral chelator Deferiprone has been approved for use as a replacement by human since 1987.^55^ In general; Deferiprone vs. Deferoxamine therapy is more economical and has shown the acceptable results in preventing complications of iron overload in patients with thalassemia major. It is currently used in Europe and Asia but it still remains without a license in North America. Because at that time, concern about the serious side effects including severe agranulocytosis has stopped further study in North America.^   55 ^^,^^56^ In order to develop the program of iron chelation therapy, Deferasirox, approved by FDA in 2005 and Canada Health Administration in 2006, is used to treat iron overload in patients next oral iron overload medication. This new drug with side effects such as renal failure and cytpenia is significantly more expensive than Desferal.^   55 ^

Although it shows more than 90% compatibility in experimental condition, patient’s compatibility is about 59% to 87%.^   56 ^ The review of available data reveals that formulation of Deferiprone as an orally active iron chelating agent has been resulted in better patient’s compliance than injectable chelators. Patient’s age is one of the factors that can lead to Deferoxamine incompatibility. 10–18 year-old age group show less consistency than others. This weak compliance can result in a significant increase in cardiovascular disease risk, endocrine disorders and death.^57^^,^^58^ The United States Health Care System uses a model to evaluate the cost effectiveness of Desferal and Deferasirox in the life quality of patient.

As a result, oral iron chelation therapy increases the cost of life as well as its standards and reduces its quality which is considered to be a challenge in choosing chelation therapy. The cost of chelation therapy not only includes its purchase but also relates to other factors such as disease symptoms control, cost of treatment, monitoring, complication, quality of life, compliance and consequences of incompatibility.^56^^,^^57^ **Importance of Chelation therapy in clinical trial**

Various studies have been shown the safety and efficiency of chelation therapy in treatment of iron overload in major beta-thalassemia patients with chronic transfusion. Since the transfusion volume affects the chelation dosage, monitoring iron intake is necessary, especially in children.^57^^,^^   58 ^ Using iron chelation therapy in transfusion-dependent anemia such as MDS, aplastic anemia and sickle cell disease has been approved. Recent reviews indicate that therapy response is dependent on chelation dosage and iron overload in body. Although the numbers of chelation therapy studies on patients with anemia due to reduction in red blood cell production such as diamond black fan are not low, there is no assessment of therapy response due to their anemia mechanism. So, there are a lot of questions still remain unanswered.^   58 ^ Various studies on the effect of iron overload due to transfusion in patients with different types of rare anemia have shown that dose of DXM and regular monitoring of serum ferritin levels are the factors involved in the control of iron.

Recently new studies have shown that DFX therapy in MDS patients can improve blood parameters and transfusion requirements. Survival statistics in MDS patients treated with DFX are acceptable.^   59 ^ The effect of iron reduction in bone marrow transplantation is diagnosed before and after transplantation. DFX has great effect on iron overload therapy in such cases. After HSCT in thalassemia patients and hematologic malignancies, iron overload is considered as an adverse prognostic factor.^   60 ^ Recent studies show the association between iron overload and liver toxicity after transplantation including chronic liver disease and susceptibility to infection. The relationship between iron overload and liver toxicity in MDS patients is significant before and after transplantation. DFX therapy effect on patient quality of life in such cases is higher than other chelation therapies.^   60 ^

Monitoring of cardiac MRI T2* values and other biomarkers of iron storage for level perception of iron overload in allogeneic hematopoietic stem cell transplantation patients are required.

Recent studies have demonstrated that there is a relationship between iron overload and pre-transplant transfusion in hematological disorders.^8^

For instance a significant difference in survival of MDS patients with the lowest and highest serum ferritin levels has been reported. Chelation therapy can effectively improve the outcomes of transplantation.^        61 ^ To date, the most studies on iron chelation therapy are in the treatment of patients with iron overload due to frequent transfusion. However, some studies have also been performed to examine other conditions of iron overload such as hereditary hemochromatosis in which iron overload progression can be seen by an increase in intestinal absorption. Cutana-tarada porphyria (a common type of Porphyria) and Mucormycosis can be associated with hemochromatosis.^        62 ^

## CONCLUSION

 Several conditions including severity of iron overload, treatment period, final costs of treatment and the results of recent studies must be taken into consideration in order to select the proper chelation therapy for a particular clinical situation. Furthermore, the additional expenses of treatment and cost of medications should be considered. Iron is also an important element in the proliferation of tumor cells. Hence, in the absence of this vital element, the proliferation of malignant cells will be also difficult.^   63 ^ Therefore, in the near future, the potential role of chelation therapy in treatment and control of cancers will be considered. Finally, the importance of knowledge and information on complete and perfect iron chelation will be revealed in the future.^   64 ^
